# Genotypic Status of the TbAT1/P2 Adenosine Transporter of *Trypanosoma brucei gambiense* Isolates from Northwestern Uganda following Melarsoprol Withdrawal

**DOI:** 10.1371/journal.pntd.0000523

**Published:** 2009-09-29

**Authors:** Anne J. N. Kazibwe, Barbara Nerima, Harry P. de Koning, Pascal Mäser, Michael P. Barrett, Enock Matovu

**Affiliations:** 1 Division of Infection and Immunity, Institute of Biomedical and Life Sciences, University of Glasgow, Glasgow, Scotland, United Kingdom; 2 Faculty of Veterinary Medicine, Makerere University, Kampala, Uganda; 3 National Livestock Resources Research Institute, Tororo, Uganda; 4 Institute of Cell Biology, University of Bern, Bern, Switzerland; 5 Wellcome Centre for Molecular Parasitology, University of Glasgow, Glasgow, Scotland, United Kingdom; Foundation for Innovative New Diagnostics (FIND), Switzerland

## Abstract

**Background:**

The development of arsenical and diamidine resistance in *Trypanosoma brucei* is associated with loss of drug uptake by the P2 purine transporter as a result of alterations in the corresponding *T. brucei* adenosine transporter 1 gene (*TbAT1*). Previously, specific *TbAT1* mutant type alleles linked to melarsoprol treatment failure were significantly more prevalent in *T. b. gambiense* from relapse patients at Omugo health centre in Arua district. Relapse rates of up to 30% prompted a shift from melarsoprol to eflornithine (α-difluoromethylornithine, DFMO) as first-line treatment at this centre. The aim of this study was to determine the status of *TbAT1* in recent isolates collected from *T. b. gambiense* sleeping sickness patients from Arua and Moyo districts in Northwestern Uganda after this shift in first-line drug choice.

**Methodology and results:**

Blood and cerebrospinal fluids of consenting patients were collected for DNA preparation and subsequent amplification. All of the 105 isolates from Omugo that we successfully analysed by PCR-RFLP possessed the *TbAT1* wild type allele. In addition, PCR/RFLP analysis was performed for 74 samples from Moyo, where melarsoprol is still the first line drug; 61 samples displayed the wild genotype while six were mutant and seven had a mixed pattern of both mutant and wild-type *TbAT1*. The melarsoprol treatment failure rate at Moyo over the same period was nine out of 101 stage II cases that were followed up at least once. Five of the relapse cases harboured mutant *TbAT1*, one had the wild type, while no amplification was achieved from the remaining three samples.

**Conclusions/significance:**

The apparent disappearance of mutant alleles at Omugo may correlate with melarsoprol withdrawal as first-line treatment. Our results suggest that melarsoprol could successfully be reintroduced following a time lag subsequent to its replacement. A field-applicable test to predict melarsoprol treatment outcome and identify patients for whom the drug can still be beneficial is clearly required. This will facilitate cost-effective management of HAT in rural resource-poor settings, given that eflornithine has a much higher logistical requirement for its application.

## Author Summary

Human African trypanosomiasis (HAT) manifests as a chronic infection caused by *Trypanosoma brucei gambiense*, or as a more acute form due to *T. b. rhodesiense*. Both manifestations occur in Uganda and melarsoprol use against the former was jeopardised in the 1990s as reports of reduced efficacy increased to the point where it was dismissed as first-line treatment at some treatment centers. Previous work to elucidate possible mechanisms leading to melarsoprol resistance pointed to a P2 type adenosine transporter known to mediate melarsoprol uptake and previously shown to be mutated in significant numbers of patients not responding to the drug. Our present findings indicate that there is a low prevalence of mutants in foci where melarsoprol relapses are infrequent. In addition we observe that at the Omugo focus where the drug was withdrawn as first line over 6 years ago, the mutant alleles have disappeared, suggesting that drug pressure is responsible for fuelling their spread. Thus constant monitoring for mutants could play a key role in cost-effective HAT management by identifying which foci can still use the less logistically demanding melarsoprol as opposed to the alternative drug eflornithine. What is required now is a simple method for identifying such mutants at the point of care, enabling practitioners to make informed prescriptions at first diagnosis.

## Introduction

Human African trypanosomiasis (HAT) or sleeping sickness is caused by *T. brucei gambiense* and *T. b. rhodesiense*. The disease affects 36 countries in sub-Saharan Africa with Angola, DR Congo, South Sudan, Uganda and Central African Republic as the most affected [1]. Current figures indicate a decline in disease incidence with an estimate of about 70,000 new cases annually compared to approximately 500,000 cases reported in the past 5 years [1]–[4]. The emergence of strains resistant to the widely used first-line drug melarsoprol has, however, negatively impacted on HAT management. An increasing incidence of melarsoprol treatment failures varying from 15%–30% has been reported in geographically distant foci in sub-Saharan Africa [5]–[8]. In 2001 this resulted in the replacement of melarsoprol with eflornithine (DFMO) as first-line therapy for late stage *T. b. gambiense* for specific foci in north western Uganda and Southern Sudan , DR Congo, and more recently in Angola.

The P2 aminopurine transporter has been implicated as a principal route of entry into trypanosomes for melamine-based arsenical drugs [9] and diamidines [10]–[13]. Loss of the transporter has consistently been shown to correlate with the emergence of drug resistance in laboratory isolates [9], [14]–[17]. Later reports demonstrated that the loss of this transporter was the result of deletion of, or point mutations in, the *TbAT1* gene (AF152369) that encodes this transporter [14],. *TbAT1* alleles from laboratory derived resistant lines and wild-type trypanosomes could be distinguished by RFLP analysis, based on changes to two *Sfa* N1 sites in the gene as a result of two specific point mutations: arginine to threonine at position 178 and asparagine to serine at position 286 [14]. The same RFLP pattern was found in *T. brucei* ssp. field isolates. A PCR/RFLP approach based on this observation was used in a study on *T. b. gambiense* isolates from Omugo in NW Uganda to demonstrate a correlation between the presence of mutant TbAT1 alleles and occurrence of relapse after melarsoprol treatment [18]. However, the observation of the *TbAT1* wild type pattern in 30% of confirmed melarsoprol refractory isolates suggested that there could be other mechanisms contributing to resistance and that the PCR-RFLP method may not detect all melarsoprol relapses [7], [18]–[20]. Nevertheless, monitoring of the status of *TbAT1* is of paramount importance since several drugs rely on the P2 transporter for import.

The increased level of treatment failures at Omugo through the 1990s led to replacement of melarsoprol with eflornithine as first line treatment for late stage HAT in this focus in 2001. In the current study we collected *T. b. gambiense*-infected blood from patients from the Omugo focus in NW Uganda, and from Moyo district, a neighbouring focus where melarsoprol is still used. We then carried out analyses using the PCR/*Sfa* NI RFLP method to determine the current status of *TbAT1* at both foci, and to show whether the *TbAT1* genotype correlates with melarsoprol treatment outcome in Moyo. Patients admitted at both sites were followed for up to 24 months post treatment for parasitological examination to ascertain treatment outcome.

## Materials and Methods

### Ethics statement

This study received ethical clearance from the Uganda National Council for Science and Technology. The protocol and consent documents were approved by the Institutional Review Boards of the National Livestock Resources Research Institute (NaLIRRI) and the Uganda National Council for Science and Technology (UNCST).

Written consent was obtained from all participants before inclusion in the study.

### Study area and population

**Figure 1:**
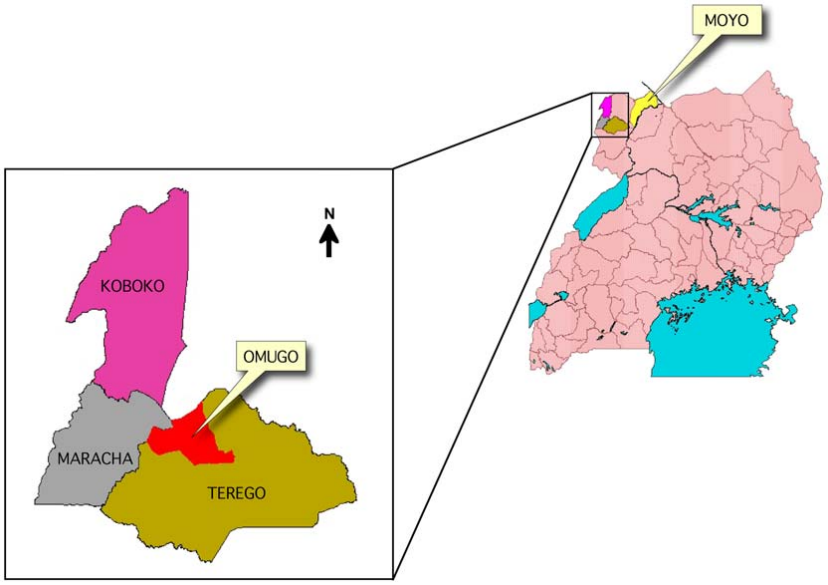
Regional Map of Uganda showing the origins (Districts/Counties) of study participants.

Field activities were carried out during the period August 2003-August 2006 in areas of the Arua and Moyo districts, in North Western Uganda (Figure 1). Arua district (latitude 2°30′N, 3°50′N and longitudes 30°30′E, 31°30′E), which has recently been sub-divided into three new districts; Arua, Terego-Maracha, and Koboko, is bordered by the Republic of Sudan in the north, DR Congo in the West, Yumbe district in the northeast, Nebbi district in the South and Gulu district in the East. Before the current break up, it was composed of six counties namely: Koboko, Maracha, Terego, Ayivu, Vurra and Madi-Okollo. Omugo health centre, the main sleeping sickness control centre, is located in Terego county (Figure 1). Moyo district (latitude 31°15′N and longitude 31°45′E) is bordered by the River Nile in the south and east, Sudan in the north, and Yumbe district in the west. It is composed of Obongi and West Moyo counties. Moyo hospital, the sleeping sickness control centre, is located in Obongi county. The main occupation in the entire study area is subsistence agriculture.

### Patient screening

Active surveillance of populations at risk of HAT was done at designated screening centres in areas of active transmission as determined from hospital records, by Mobile teams aided by Sleeping Sickness Assistants (SSAs) and local leaders. Screening of patients was done according to an established diagnostic tree in use in northwest Uganda. All presenting persons were screened for trypanosome antibodies with the Card Agglutination Test for Trypanosomiasis (CATT). Antibody levels in these potential HAT cases were also determined using 1/4 and 1/8 dilutions of blood plasma for the individuals positive in the CATT whole blood test. Persons found positive after plasma dilution were subjected to parasitological diagnostic procedures as below.

### Parasitological diagnosis

Parasitological methods for detection of trypanosomes were carried out at the respective health units (Omugo Health Centre and Moyo Hospital). Wet smears of gland aspirates from individuals with swollen lymph nodes were examined for motile trypanosomes under the microscope at 40× magnification. In addition, blood was collected by venipuncture into heparinised vacutainer tubes and subjected to the Haematocrit Centrifugation Technique (HCT) [21]. Presence of trypanosomes in the lymph aspirates or blood was confirmation that the sero-reactive individual was indeed a HAT case. A lumbar puncture was performed for collection of cerebrospinal fluid (CSF) to check for central nervous system (CNS) invasion by double centrifugation [22]. Prior to the centrifugation, a 50 µl aliquot of CSF was used on the Rosenthal chamber to estimate the white blood cell count (WBC). Patients with demonstrated presence of trypanosomes in CSF or with an elevated WBC >5/mm^3^ were considered to be late stage cases.

Individuals who were sero-reactive up to CATT 1/8 plasma dilution but were parasitologically negative were asked to report for re-testing after 3 months. All confirmed HAT individuals were hospitalized for the duration of treatment. Those who consented after description of the study were enrolled.

### Treatment

Stage I patients at both health units were treated with pentamidine isethionate (4 mg/kg body weight/day) for 10 days administered intramuscularly. Stage II patients in Moyo were treated with a melarsoprol schedule of 3 series of 4 daily injections, each series separated by 7 days. In Omugo, eflornithine (DFMO; 400 mg/kg body weight/day) was administered intravenously with 4 daily infusions (100 mg/kg body weight every 6 hours) for 14 days. Upon completion of treatment, patients were discharged and requested to return after 6, 12, and 24 months to monitor for treatment success. Patients showing signs of infection by microscopy or elevated cell white cell counts during follow up at Moyo hospital were re-admitted and treated with DFMO.

### Collection of trypanosome samples

Whole blood and/or CSF for molecular analysis were collected within 2 hours of written consent, prior to treatment. A total of 222 patients were enrolled into this study: 121 from Moyo and 101 from Arua, including 52 Koboko, 24 from Terego, 6 from Maracha, 5 from Madi-Okollo counties and 14 from the neighbouring Yumbe district.

For each HAT case enrolled in the study, samples were collected both as blood spotted on FTA cards and as whole blood and/or CSF. Two ml of whole blood from each individual collected by venipuncture was spotted (500 µl per spot) onto an FTA card (Whatman), air dried and stored in a self-sealing plastic bag at room temperature for later molecular analysis. Trypanosome genomic DNA was extracted from whole blood and CSF samples using a commercial kit (PUREGENE, Gentra Systems, Minneapolis, USA) according to the manufacturer's instructions. In this form easy storage at room temperature was possible to await transportation to the laboratory for completion of DNA extraction, performed exactly as described [18] within 2 weeks of addition of the sample to the cell lysis solution of the kit.

### PCR amplification of *TbAT1* 677 bp fragment

Trypanosome DNA from a 2.0 mm disc punched from an FTA card was extracted using the FTA purification reagent following the manufacturer's instructions (www.whatman.com/repository/documents/s7/FTAProtocolBook602.pdf). The *TbAT1* fragment for RFLP analysis was amplified from extracted DNA by nested PCR [18]. In the primary PCR, each DNA disc or 5 µl of genomic DNA solution purified from whole blood/CSF was amplified in a total volume of 25 µl PCR reaction containing a 1× custom PCR master mix (Advanced Biotechnologies Ltd, UK). The oligonucleotide primer pair TbAT1 ant-s (5′-GCC CGG ATC CGG CTG GTT TTT AGA CAA AAG TGA T-3′) and TbAT1 ant-as (5′-GCC CCT CGA GCC GCA TGG AGT AAG TCT GA-3′) [14] and 1.25 units of Taq polymerase (Promega) were added to the reaction performed in a PTC-100 DNA engine (MJ Research, Waltham, MA, USA) under the following conditions: initial denaturation at 94°C for 4 minutes; denaturation at 94°C for 30 seconds, annealing at 50°C for 30 seconds, and extension at 72°C for 1 minute for 30 cycles with a final extension at 72°C for 7 minutes. Genomic DNA of *T. brucei* strain STIB 950 (a reference clone isolated from a bovine in Somalia, resistant to berenil, isometamidium and quinapyramine) [23] and sterile water (no template) were included in each run as a positive and negative control respectively. 2 µl of primary PCR product was used as template in a secondary PCR with oligonucleotide primer pair ATF-2 (5′-CGC CGC ACT CAT CGC CCC GTT-3′) and ATR-2 (5′-CCA CCG CGG TGA GAC GTG AT-3′) under similar conditions but the annealing temperature was increased to 65°C [18]. Ten micro litres of each secondary PCR product were analysed by electrophoresis on a 1% agarose gel stained with ethidium bromide (0.7 µg/ml) and visualised under ultraviolet transilluminiscence. The band sizes were determined by comparison with a standard 1-Kb molecular weight DNA marker (Gene Ruler, Fermentas Life Sciences).

### Whole genome amplification

To increase the PCR sensitivity of the DNA samples on FTA cards (Whatman) and genomic DNA from whole blood or CSF, the whole genome amplification method was carried out on selected samples as described [24] using a commercial kit (GenomiPhi, Amersham Biosciences). Reactions were carried out in triplicate for each individual sample following the manufacturer's instructions. Briefly, a DNA sample was denatured prior to amplification. To a DNA sample on an FTA card disc was added 10 µl of sample buffer in a sterile microfuge tube, which was heated to 95°C for 3 minutes in a heating block and then cooled to 4°C on ice. Alternatively, to 1 µl of DNA sample from whole blood or CSF (at least 1 ng/µl) was added 9 µl of sample buffer. For each amplification reaction, 9 µl of reaction buffer was combined with 1 µl of enzyme mix on ice added to the cooled sample. The sample was then incubated at 30°C in a heating block for 16–18 hours. Following the overnight incubation, the sample was heated to 65°C for 10 minutes in a heating block to inactivate the enzyme and immediately cooled to 4°C. The 3 separate reactions for each individual sample were pooled. 1–2 µl of whole genome amplified DNA was ready for use in PCR or kept at −20°C.

### RFLP using *Sfa* NI

For each isolate with a successfully amplified *TbAT1* 677 bp fragment, 5 µl of PCR product was digested with the *Sfa* NI enzyme to determine the *TbAT1* genotype [14],[18]. The reaction was carried out in a total volume of 20 µl containing NEBuffer 3 and 1 unit of *Sfa* NI enzyme (New England Biolabs). The reactions were incubated at 37°C overnight and then analysed by gel electrophoresis on a 2.5% Agarose gel stained with ethidium bromide. The generated fragments were visualised under Ultraviolet trans-illumination.

## Results

### *TbAT1* amplification

**Figure 2:**
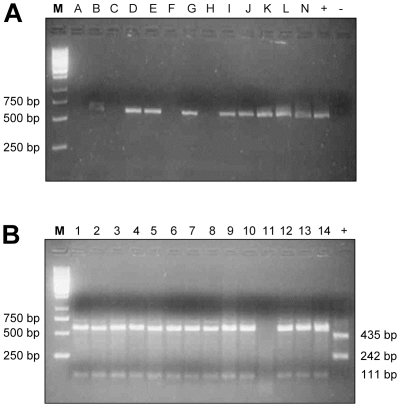
Electrophoretic analysis of PCR-RFLP products. Representative Agarose gel results (A) *TbAT1* 677 bp PCR product of the trypanosome isolates from patients at Omugo (lanes B,D,E,G,I,J,K and N). The positive control (+) is a multidrug resistant clone STIB 950, negative control (−) was sterile water, while M is a 1 Kb molecular marker (Gene rulerTM, Fermentas, Life Sciences). (B) *Sfa NI* digest of the isolates from Omugo *TbAT1* 677 bp fragments. *TbAT1* wild type pattern of 566 bp and 111 bp fragments was displayed by all isolates from Omugo (lanes 1–10, 12–14), while the positive control (STIB 950) displayed the mutant genotype of 435 bp and 242 bp.

**Figure 3:**
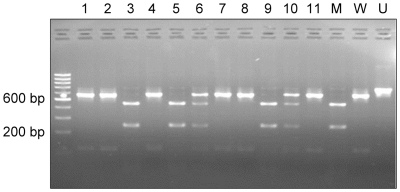
PCR/RFLP patterns of selected samples from Moyo. U = undigested 677 bp fragment; W = wildtype pattern (STIB777S); M = Mutant (STIB777R); samples 1–11 are from patients M058, M052, M106, M048, M103, M074, M047, M044, M069, M062, and M031 respectively. Note the mutant pattern displayed in lanes 3, 5, 9, and the mixed genotype in lanes 6 and 10.

Given logistical problems in collecting and processing infected blood for PCR analysis [24],[25], we used Whatman FTA cards to collect blood spots from infected patients in the two foci, Omugo and Moyo. In pilot experiments (data not shown) using infected rodent blood we showed that the TBR multicopy PCR primers can detect trypanosomes from blood down to a density of fewer than 1 parasite per ml. With single copy genes such as *TbAT1*, however, the limit of detection was 10^4^ trypanosomes per ml of blood (data not shown). From a total of 222 DNA samples from patient blood and/or CSF, amplification of the *TbAT1* 677 bp fragment by nested PCR was successful for 179 (80%) (Figs 2, 3). Presence of trypanosomal DNA in 34 of the samples in which *TbAT1* PCR had failed was determined by PCR using the highly sensitive *T. brucei* multi-copy locus primers TBR-F and TBR-R [26] on a subset of 133 samples including 65 samples that were already *TbAT1* positive. Results revealed trypanosome DNA positive samples for all except one sample of patient M169 from Moyo indicating a possible absence of *T. brucei* DNA in that sample. In order to increase the starting DNA content before amplification of the single copy *TbAT1*, a whole genome amplification kit (GenomiPhi, Amersham Biosciences) was used on the problematic samples. Of the 68 initially *TbAT1* PCR failed DNA samples, 26 were successfully re-amplified for *TbAT1* (n = 17 Omugo and n = 9 Moyo) using this approach, bringing the total of *TbAT1* successfully amplified samples to 179/222.

The entire 133 sample sub-set was also PCR amplified for the *T. brucei* single copy Phospholipase C (PLC) associated microsatellite. This was successful for 61 (45.8%) of the isolates. Thus, the PCR success rate of the TBR multicopy locus was higher than that of *TbAT1*, which was in turn higher than that of the single copy PLC microsatellite. These results strongly suggest that the PCR failed samples had a low DNA content below detection threshold for *TbAT1* in 42 isolates and for the single copy *PLC* microsatellite in 72 isolates, probably due to low parasite densities. Attempts with alternative unpublished primer sets that we routinely use to amplify different *TbAT1* fragments did not yield any signals from these samples. However, complete loss or deletions in *TbAT1* – as was shown in some laboratory strains [27],[28] and at least one field isolate from Angola (stock K001) [18] – cannot be ruled out, especially for the samples that failed PCR for *TbAT1* but were successfully amplified for the single copy *PLC* microsatellite (n = 5 Omugo and n = 7 Moyo).

### PCR/*Sfa* NI RFLP analysis for TbAT1 genotype

The analysis of the *TbAT1* 677 bp fragment for each of the 91 isolates from Omugo by digestion with *Sfa* NI revealed exclusively the wild type banding pattern (566 bp and 111 bp; Fig 2) identical to the pattern displayed by the melarsoprol-sensitive *T. brucei* stock STIB 777S [14]. This suggested the patients were infected with trypanosomes of *TbAT1* ^+/+^ wild type genotype, an indication that they were melarsoprol-sensitive trypanosomes. Successful analysis of 74 samples from Moyo, where melarsoprol is still the first line drug, revealed that 61 displayed the wild-type genotype while 6 were homozygous for the mutant allele and 7 had a mixed pattern of both mutant and wild-type *TbAT1* (see Fig 3 for representative patterns).

### Patient follow up for determination of treatment outcome

Pentamidine isethionate was used for treatment of Stage I patients at both health units, whereas stage II HAT was treated with melarsoprol in Moyo and with eflornithine in Omugo (see Materials and Methods).

Follow-up was performed according to established National program guidelines at 6, 12 and 24 months. A total of 188 out of the 222 (85%) patients included in this study were followed up at least once. Of these, 7/31 (2 from Omugo and 5 from Moyo) were re-admitted 6–24 months after Pentamidine treatment and were confirmed as stage II cases as demonstrated by the presence of trypanosomes in CSF (5/7) coupled with elevated white cell counts (7/7) compared to when they were first admitted. *TbAT1* genotyping was only possible for 2 samples from Moyo and showed mixed and wild-type patterns, respectively. Given the known irrelevance of *TbAT1* to DFMO (eflornithine) treatment outcome, follow up results for this drug are beyond the scope of this paper and will be discussed elsewhere (manuscript in preparation).

Melarsoprol relapses could only be investigated at Moyo where the drug is still first-line. Out of 101 samples collected, 61 were wild type, 7 mutant and 3 had mixed genotype, while no amplification was possible from the remaining 27. Nine out of the 101 (9%) stage II patients were readmitted 6–12 months post-treatment, with trypanosomes in the CSF and cell counts ranging 7–248/mm^3^ (Table 1). In 6 of the relapses, the white cell counts had declined from those at initial admission although trypanosomes persisted within the CSF. *TbAT1* analysis of DNA samples recovered from the CSF revealed that 5 were mutant and 1 possessed the wild-type. No signals were obtained from the remaining 3 samples, probably due to limiting amounts of parasite DNA therein. Assuming that the mixed allelotypes observed were either heterozygotes or mixed infections of equal numbers of homozygous wild type and homozygous mutants, the overall gene frequencies are 0.865 (122+6/148) for *wt* and 0.135 (14+6/148) for *mut*. It would therefore follow that the probability *P* of having by chance at least 5 out of 6 melarsoprol relapse samples as *mut* (as was observed in this study), is 0.00024. Thus it is highly significant to have 5 *mut* patterns in the relapse group, a further evidence to incriminate *TbAT1* involvement in melarsoprol relapse.

**Table 1:** Melarsoprol relapse patients at Moyo Hospital and the *TbAT1* genotypes in samples recovered from their CSF.

Code	Initial CSF Cells/Tryps	Relapse after	CSF Cells/Tryps	*TbAT1* genotype
M048	74/+	06 months	07/+	Wildtype
M059	240/+	06 months	104/+	Mutant
M064	210/+	06 months	158/+	Mutant
M073	56/+	06 months	132/+	Mutant
M094	36/+	06 months	42/+	Failed PCR
M103	240/+	06 months	84/+	Mutant
M106	210/−	06 months	158/+	Mutant
M139	41/−	12 months	248/+	Failed PCR
M141	36/+	06 months	42/+	Failed PCR

Tryps = trypanosomes; + = Trypanosomes present; − = Trypanosomes not observed. CSF cells counts are indicated as cells/µl.

## Discussion

While mutations in *TbAT1* have clearly been implicated in drug resistance of *T. b. brucei* in the lab, the role of *TbAT1* in treatment failures with *T. b. gambiense* in the field is unclear. It was recently shown in Southern Sudan, where melarsoprol had also been replaced with eflornithine in 2001, that trypanosomes in circulation in 2003 only had the so called wild type alleles at the *Sfa* N1 RFLP sites [29]. This led the authors to conclude that it was unlikely that this resistance allele had been in circulation in 2001, and that it was possible that reasons other than loss of P2 activity had been responsible for the melarsoprol treatment failures there. However, that study could not rule out the possibility that removal of melarsoprol as first line treatment led to loss of a selective pressure on circulating trypanosomes and that a fitness cost associated with mutant *TbAT1* alleles led to loss of those alleles from the population. There was no base-line data available on genotypes found in Southern Sudan when melarsoprol was in use to address this hypothesis.

Given that data was available for allelotypes in Omugo during the time when melarsoprol treatment failure was prevalent (see Table 2), it was of considerable interest to determine whether the so-called mutant allele that was prevalent at that time was still present several years after melarsoprol selection pressure had been withdrawn. We also generated allelotype data from a second site in Northern Uganda (Moyo) where melarsoprol has remained in use until today.

**Table 2:** Melarsoprol relapse rates and *TbAT1* genetic status, 1998 to present study.

	Omugo 1998^a^	Omugo by 2006	Moyo by 2006
Stage II patients	65	90	101
Melarsoprol relapses	43	n.a.^b^	9
Frequency of *TbAT1* mutant alleles:			
i) - in newly infected patients	36%	0%	12%
ii) - in relapse patients	70%	n.a.^b^	83%
**Total**	58%	0%	18%

a Data from [13].

b Use of melarsoprol discontinued in 2001.

The *Sfa* N1 RFLP profile previously reported [14],[18],[20] comprises either a pattern yielding bands of 566 bp and 111 bp in wild-type parasites, or of 435 bp and 242 bp in the resistant parasites. Of the 105 samples from Omugo where melarsoprol has not been used since 2001, only the wild type pattern was observed in specimens from which *TbAT1* did amplify. By contrast, at Moyo, where melarsoprol is still in use, of 74 amplified *TbAT1* samples, 61 isolates displayed the wild-type pattern, 6 had the mutant (resistant) pattern and seven showed a mixed pattern.

To determine if a difference existed between the above *TbAT1* results obtained from Omugo and Moyo, the samples with the observed *TbAT1* allele types were grouped separately or combined and analysed using the Chi squared test. The results showed a significant difference (*χ* ^2^ = 19.9, P = 0.000 and *χ* ^2^ = 18.1, P = 0.000 respectively). This suggests a significant difference in allelotype compositions at the two sites, namely the presence of *TbAT1* mutant alleles in Moyo and their absence in Omugo.

Our results seem to suggest that the P2 transporter gene, in its mutant form, actually carries a fitness cost such that mutants could disappear from a locality once the selective pressure exerted by melarsoprol is removed (see Table 2 for prevalences of mutant genotypes at two time points, before and after melarsoprol withdrawal).. Interestingly, no fitness cost to the absence of a functional TbAT1 transporter was observed under *in vitro* laboratory conditions; on the contrary, *tbat1^−/−^* null mutant trypanosomes grew even faster than *TbAT1^+/+^* parasites *in vitro* [30] and the *tbat1^−/−^* parasites were as infective as the parental line to laboratory rodents [15]. Definitive proof is lacking, but the disappearance of this allele from Omugo has coincided with the removal of melarsoprol as first line treatment. At the same time, the persistence of *TbAT1* mutant alleles at Moyo, where melarsoprol is still in use, and the correlation between *TbAT1* mutant alleles and melarsoprol treatment failure, would also support this hypothesis.

If it is the case that removal of melarsoprol selection pressure is associated with loss of mutant *TbAT1* alleles that contribute to drug resistance, this would indicate that melarsoprol could eventually be suitable for treatment in many areas where failures have led to its withdrawal. In order to achieve this, it would be necessary to make a rapid diagnosis of the status of the P2 transporter in parasites from patients in a given focus. This could be achieved, for example, using the fluorescence test for melarsoprol resistance [31]. Application of tests such as this could lead to a rational choice of treatment with melarsoprol, or eflornithine. Indeed given its toxicity, melarsoprol can not be the drug of choice; its continued use could nevertheless delay resistance to the latter that is likely to appear fast due to its being just a trypanostatic drug. Coupled to alternate use of the two drugs, the nifurtimox-eflornithine combinations currently under consideration [32] would minimise the prospects of treatment failure and concomitantly reduce the risk of spreading drug resistance.
